# The mechanism of low molecular weight fucoidan-incorporated nanofiber scaffolds inhibiting oral leukoplakia via SR-A/Wnt signal axis

**DOI:** 10.3389/fphar.2024.1397761

**Published:** 2024-07-22

**Authors:** Ming Xu, Yu Sun, Beibei Cong, Xiaopei Zhang, Zhenfeng Li, Yingnan Liu, Lihua Geng, Qi Qin, Yingtao Wu, Meihua Gao, Wanchun Wang, Yuanfei Wang, Yingjie Xu

**Affiliations:** ^1^ Qingdao Medical College, Qingdao University, Qingdao, China; ^2^ Qingdao Stomatological Hospital Affiliated to Qingdao University, Qingdao, China; ^3^ Institute of Neuroregeneration & Neurorehabilitation, Department of Pathophysiology, School of Basic Medicine, Qingdao Medical College, Qingdao University, Qingdao, China; ^4^ Experimental Center for Medical Research, Weifang Medical University, Weifang, China; ^5^ CAS and Shandong Province Key Laboratory of Experimental Marine Biology, Center for Ocean Mega-Science, Institute of Oceanology, Chinese Academy of Sciences, Qingdao, China

**Keywords:** fucoidan, electrospun nanofiber, oral leukoplakia, scavenger receptor A, Wnt/β-catenin

## Abstract

Oral leukoplakia (OLK) is the most common oral precancerous lesion, and 3%–17% of OLK patients progress to oral squamous cell carcinoma. OLK is susceptible to recurrence and has no effective treatment. However, conventional drugs have significant side effects and limitations. Therefore, it is important to identify drugs that target OLK. In this study, scavenger receptor A (SR-A) was found to be abnormally highly expressed in the oral mucosal epithelial cells of OLK patients, whereas molecular biology studies revealed that low molecular weight fucoidan (LMWF) promoted apoptosis of dysplastic oral keratinocytes (DOK) and inhibited the growth and migration of DOK, and the inhibitory effect of LMWF on OLK was achieved by regulating the SR-A/Wnt signaling axis and related genes. Based on the above results and the special situation of the oral environment, we constructed LMWF/poly(caprolactone-co-lactide) nanofiber membranes with different structures for the *in-situ* treatment of OLK using electrospinning technology. The results showed that the nanofiber membranes with a shell-core structure had the best physicochemical properties, biocompatibility, and therapeutic effect, which optimized the LMWF drug delivery and ensured the effective concentration of the drug at the target point, thus achieving precise treatment of local lesions in the oral cavity. This has potential application value in inhibiting the development of OLK.

## 1 Introduction

Oral leukoplakia (OLK) is the most common precancerous oral cavity lesion, predominantly affecting middle-aged and older adults (Farah, 2021). Studies have shown that OLK has malignant potential, with 3%–17% of patients developing oral squamous cell carcinoma (OSCC) (Weber et al., 2020). Cures for OLK, such as exfoliating medicines and surgery, have limitations and may result in traumatic surfaces. Therefore, it is necessary to design a medication that targets OLK and suppresses its development without side effects. Research has demonstrated that the polarization and infiltration of macrophages are necessary for OLK development (Weber et al., 2020). As a unique receptor on the surface of macrophages, the scavenger receptor (SR) plays a significant role in lipid metabolism and the pathological processes involving macrophages. However, the effects of the SR on oral diseases require further investigation. SR-A, a class A SR, triggers the host’s innate immune response by identifying the chemical patterns of various pathogens (Xiang et al., 2020). SR-A is a possible molecular marker of rheumatoid arthritis that contributes to its development (Hu et al., 2020). Therefore, SR-A may serve as a molecular marker and therapeutic target for several clinical disorders. Our previous study revealed that patients with OLK exhibited a significant quantity of SR-A-positive expression in all layers of epithelial cells compared to the normal human oral epithelium. Therefore, we hypothesized that SR-A plays a role in the malignant transformation of the oral epithelium, thereby affecting and promoting the development of OLK. Identifying an SR-A ligand analog to prevent SR-A expression may be a new approach for the treatment of OLK.

Laminaria japonica has long been used in Chinese medicine, and fucoidan, a natural extract of Laminaria japonica, has been applied in many fields (Lee et al., 2017; Fitton et al., 2019). The biological activity of fucoidan varies with the degree of sulfation, molecular weight, sulfation mode, and glycosidic branching. Owing to its distinctive structure, it is a natural ligand for several proteins, including SR-A and selectin. It exhibits various biological activities, including anti-inflammatory, antioxidant, and anti-tumor activities (Yang et al., 2022). Studies have shown that low molecular weight fucoidan (LMWF) can bind SR-A targeting to control macrophage immunological activity and other associated consequences, thus curing clinical disorders (Nishinaka et al., 2020; Zhu X. et al., 2021; Cui et al., 2022; Sun et al., 2022) and altering the expression of several signaling pathways (Boo et al., 2013; Xu et al., 2019). One of these significant signaling pathways, the Wnt/β-catenin signaling pathway, is crucial in oral disorders. Oral mucosal diseases can develop into malignancies because of the aberrant activation of the Wnt pathway (Liu and Millar, 2010). In relevant research, Wnt3, β-catenin, and cyclin D1 have all been found in OLK tissues, indicating that the Wnt pathway may be implicated in OLK development (Ishida et al., 2007).

Due to the unique oral environment, variables such as saliva and food affect the therapeutic effect of medications on oral mucosal lesions. LMWF is significantly diluted in the oral cavity because it is a water-soluble heteropolysaccharide, and traditional therapies such as oral administration and topical application cannot ensure an effective drug concentration at the lesion. Therefore, there is an urgent need to develop a novel oral mucosal drug delivery system to improve the drug-loading pathway of LMWF and enhance its efficiency in the oral mucosa. Nanomaterials have been widely used as medicinal materials in recent years owing to their superior physical qualities and drug-carrying capabilities. Poly(caprolactone-co-lactide) (PLCL) has excellent biocompatibility, mechanical characteristics, and degradation properties (Li L. et al., 2022). It was discovered that PLCL/Gelatin/epigallocatechin gallate (EGCG)/core–shell nanofiber membranes composed of PLCL-loaded EGCG could maintain medication release while promoting wound healing (Li A. et al., 2022). However, the use of PLCL composite biomaterials for the treatment of oral disorders has been insufficiently described. This study aimed to investigate the molecular mechanism by which LMWF inhibits the development of OLK and to use different electrospinning techniques and a series of physicochemical characterizations to prepare and screen novel LMWF/PLCL nanofibrous membranes to optimize oral mucosal drug delivery and achieve precise topical drug delivery. As a result, functionalized nanofiber PLCL loaded with marine polysaccharide LMWF can be employed as a biological patch to provide new concepts and methods for LMWF-targeted therapy of OLK lesions.

## 2 materials and methods

### 2.1 Materials

LMWF (MW = 8,177 Da) was provided by the Institute of Oceanography, Chinese Academy of Sciences (Qingdao, China). Laminaria japonica cultured in Rongcheng (Shandong, China) was collected, washed, transported to the laboratory, and extracted using hot water distillation (Wang et al., 2009). The extracted LMWF was analyzed using high-performance liquid chromatography, capillary electrophoresis, monosaccharide composition analysis, methylation analysis, periodate oxidation, and Smith degradation.

The following reagents were used: RNAi-interfering lentivirus (LV-EGFP-RNAi) and negative control virus (LveGFP) were constructed by Gikai Gene Chemistry Technology Ltd. (Shanghai, China); CCK-8 kit was purchased from GLPBIO Co., Ltd. (Montclair, CA, United States); Annexin-V-FITC/PI Apoptosis Kit and Annexin V-APC/PI Apoptosis Kit were purchased from Wuhan Jingrui Biotechnology Co., Ltd. (Wuhan, Hubei, China); TRIzol kit was purchased from Sigma-Aldrich Co., Ltd. (St. Louis, Missouri, United States); SYBR PreMix Ex TaqTMII, PrimeScriptTM RT kits were purchased from Takara Biotech Co., Ltd. (Kusatsu, Shiga, Japan); Primers were designed and synthesized by Shanghai Biotechnology Co; β-catenin, TCF4, and Frizzled 6 antibodies were purchased from Abcam (Cambridge, United Kingdom); β-actin, SR-A antibodies was purchased from Proteintech (Chicago, United States); AXIN1 antibody was purchased from Cell Signaling Technology (Danvers, MA, United States); Artificial saliva and Wnt/β-catenin signaling pathway inhibitor IWR-1 were purchased from MedChemExpress (Monmouth County, New Jersey, United States); PLCL (50:50, Mn:300000) was purchased from Shenzhen Maiqi Biomaterials Co; Hexafluoroisopropanol (HFIP) was purchased from Shanghai Darui Fine Chemical Co; DMMB Taylor’s Blue was purchased from Shanghai Maclean Biochemical Technology Co; Poly-L-lysine Solution was purchased from Beijing Solexpro Technology Co; L-polylysine (PLL) was purchased from Thermo Fisher Scientific (Massachusetts, United States).

### 2.2 Patients and clinical specimens

Thirty clinical samples of OLK were chosen from the Oral Mucosa Department of Qingdao Stomatological Hospital, and 10 samples of healthy oral mucosa were used as controls (normal group). Samples from the OLK group were obtained from patients with pathologically proven OLK with HE staining and clinical presentation. None of the patients had ever undergone relevant therapy, and healthy oral mucosal tissues were obtained from patients who have undertaken tooth extraction, implant placement, or orthognathic surgery (Warnakulasuriya et al., 2007). Based on the World Health Organization (2005) criteria (Warnakulasuriya et al., 2021), OLK histology was categorized as mild, moderate, or severe aberrant hyperplasia. The Medical Ethics Committee of Qingdao Stomatological Hospital approved this research project (2021KQYX032). Patients and their families completed the informed consent forms, and all studies were conducted in accordance with the Declaration of Helsinki.

### 2.3 Immunohistochemistry

OLK lesion tissues were collected, embedded in paraffin, and sectioned at a thickness of 4 μm for immunohistochemical staining. Five fields of view were randomly selected for each image, and brownish-yellow granules were counted as positive cells. Image-Pro Plus detected positive SR-A levels, and these values were expressed as the average optical density (AOD).

### 2.4 Cell culture

The Wuhan University School of Medicine provided dysplastic oral keratinocyte (DOK) cells and human oral keratinocyte (HOK) cells. The HOK cells were cultured in a high-glucose DMEM medium containing 10% fetal bovine serum and 1% Penicillin-Streptomycin double antibiotic. The DOK cells were cultured in a RPMI 1640 medium containing 10% fetal bovine serum and 1% Penicillin-Streptomycin double antibiotic. All cells were cultured at 37°C, 5% CO_2_, and 70%–80% RH. Cells in the logarithmic growth phase were used for subsequent experiments.

### 2.5 Lentivirus transfection

The DOK cells were seeded overnight in 24-well plates at a density of 1 × 10^4^ cells/well, and 500 μL of lentivirus dilution was added to each well according to the multiplicity of infection (MOI = 30) and virus titer (1 × 10^8^ TU/mL). The cells were divided into three groups: control, negative empty vector (LVGFP), and SR-A knockout (LV-GFP-SR-A RNAi). The transfection efficiency was evaluated after 72 h using the fluorescence microscope CKX53 and further screened with puromycin for subsequent experiments.

### 2.6 CCK-8 analysis

The DOK cells were seeded in 96-well plates at 0.5 × 10^4^ cells/well. The groups were incubated overnight for 24 h, according to the manufacturer’s instructions. The absorbance of each group (OD) was then measured at 450 nm using an 800TS enzyme labeler.

### 2.7 Colony formation assay

Cells from different groups were seeded in 6-well plates at 1 × 10^3^ cells/well density and incubated for 14 d. Next, the cells were washed three times with phosphate-buffered saline (PBS), fixed in 100% methanol for 30 min, and stained with 1% crystal violet for 30 s. Three areas were randomly selected and observed via microscopy.

### 2.8 Cell apoptosis

Cells from different groups were collected separately, centrifuged at 1,000 rpm for 5 min, and washed once with PBS. 500 μL 1×Annexin V Binding Buffer working solution was added and resuspended, and then 5 μL Annexin V-FITC and 5 μL propidium iodide (PI) were added for staining, followed by incubation for 15–20 min at room temperature in the dark. The apoptosis rates were measured using a CytoFLEX flow cytometer.

### 2.9 Wound healing assay

Cells were seeded in 6-well plates at a density of 5 × 10^4^ cells/well and cultured until the cells were spread evenly over the well plates. Scratches were marked perpendicular to the bottom of the well plate and washed with PBS. The cells were then incubated with low serum (<2%) RPMI1640 complete culture medium for 12 h. Records were taken at 0 and 12 h. Scratch photographs of the different groups were observed and analyzed. The mean values of the distances between cells were calculated using ImageJ software, and all measurements were repeated three times.

$$Wound\;healing\;rate\left(\%\right)=\frac{\left(A0-A1\right)}{A0}\times 100$$ (1)

In the formula, A0 is the width value of the initial scratches; A1 is the width value of current scratches.

### 2.10 RNA extraction and high-throughput sequencing

The total RNA was extracted from the treated cells using TRIzol, and the RNA concentration was measured using a Micro Drop ultra-micro spectrophotometer. Relevant transcriptome sequencing was performed by Beijing Allwegene Technology Co.

### 2.11 RT-qPCR analysis

RNA was extracted as described above. cDNA was synthesized using a reverse transcription kit. All samples used GAPDH as an internal reference, and the results were relatively quantified using the 2-^△△^Ct method. The primers used were SR-A: 5′-TAG GCA CTT GGG ATG TCT GA-3′ (forward) and 5′-GTC CTC AAT TTG TAT TGG TGC T-3′ (reverse); CTNNB1:5′-GAG GAG ATG TAC ATT CAG CAG A-3′ (forward) and 5′-GTT GAC CAC CCC TGC ATA G-3′ (reverse); AXIN1:5′-TGG ATG ACC AAG ATG GGA TAA G-3′ (forward) and 5′-GAC ACG ATG CCA TTG TTA TCA A-3′ (reverse); FZD6: 5′-GCC ACT GTG CCT TTG TGT GTT TG-3′ (forward) and 5′-AAG CCG CTG AAG ACT CCA ATT CG-3′ (reverse); TCF4: 5′-TCC AGT CTT CCT CCG ATG TCC AC-3′ (forward) and 5′-GCT GCC CCG CTT CCT CTA TTT G-3′ (reverse); CTNNBIP1: 5′-TGC TGC GGA AGA TGG GAT CAA AC-3′ (forward) and 5′-CTG GCT GAG CTG GCT GTT GAC-3′ (reverse).

### 2.12 Western blot assay (WB)

The total protein from the different groups was extracted using a RIPA lysis buffer, and the protein concentration was detected using a BCA kit. SDS-PAGE was performed, and the protein was electro-transferred to a polyvinylidene fluoride (PVDF) membrane. It was blocked with 5% skim milk and incubated for 2 h at room temperature and with primary antibody incubation overnight at 4°C. After washing with TBST, HRP-labeled protein bands were visualized using an ECL kit. The bands were analyzed using ImageJ software with β-actin as an internal reference.

### 2.13 Preparation and characterization of LMWF/PLCL nanofiber membranes

#### 2.13.1 Preparation of nanofiber membranes

Blended nanofiber membranes: Configuring 10% PLCL solution, LMWF water solution was added to the spinning solution with a final concentration of 50 ug/mL, and the blended nanofiber membranes were prepared *via* uniaxial electrospinning.

Shell-core nanofiber membranes: By setting up 10% PLCL solution as the shell layer solution and 50 μg/mL LMWF as the core layer solution, with the flow rates of the solutions set to 1.0 mL/h and 0.8 mL/h, respectively, the shell-core structured nanofiber membranes were created *via* coaxial electrospinning.

Coated nanofiber membrane: 10% PLCL nanofibrous membrane was prepared by uniaxial electrospinning, plasma treating the surface of the fibrous membrane for 2 min, immersion in 0.1% PLL solution for 2 h, rinsing with PBS, and then immersion in 50 μg/mL LMWF aqueous solution at 4°C overnight. Finally, it was dried.

Blank nanofiber membrane: PLCL nanofiber membranes were prepared *via* uniaxial electrospinning using a 10% PLCL solution.

The above electrospinning process was set to 12 kV, the flow rate was set to 1.0 mL/h, and the receiving distance was 15 cm, and it was performed at a room temperature of 25°C and ambient humidity of 40%. The prepared fiber membranes were stored at −20°C.

#### 2.13.2 Scanning electron microscopy (SEM)

The nanofiber membranes were cut into 1 cm × 1 cm samples for gold spraying, and their morphologies were observed using scanning electron microscopy (SEM). Fifty randomly selected nanofibers were used to measure the diameter using the ImageJ software. The diameter data were analyzed using Origin 9.0, and the diameter distribution was plotted.

#### 2.13.3 Transmission electron microscope (TEM)\

The electrospun nanofibers were sprayed onto a copper mesh. After the organic solvent evaporated, the structure of the nanofiber membrane was examined using TEM at an operating voltage of 100 kV.

#### 2.13.4 Fourier transform infrared spectroscopy (FTIR)

The Fourier transform infrared spectroscopy (FTIR) spectra of the samples in the range 500–4,000 cm^−1^ were characterized using a Nicolet iN10 FTIR spectrometer with a scan resolution of 2 cm^−1^ and 32 scans. The results were analyzed using Origin 9.0.

#### 2.13.5 Water contact angle (WCA)

The water contact angle (WCA) of the nanofiber membrane was determined using a contact angle analyzer. After 5 μL deionized water was placed over the nanofiber membrane, which had been cut into a square with a surface area of approximately 1 cm^2^, the angle between the tangent of the water droplet and the nanofiber membrane was measured after 10 s. This procedure was repeated for each sample at three different locations, and the mean and standard deviation of the WCA for each sample were calculated.

#### 2.13.6 Mechanical strength of the nanofiber membrane

A universal material testing machine was used to evaluate the mechanical properties of the nanofiber membranes. The nanofiber membranes were cut into 1 cm × 5 cm rectangular samples and tested at a tensile speed of 50 mm/min; the tensile data were recorded and analyzed.

#### 2.13.7 Drug loading efficiency and encapsulation efficiency

The DMMB approach was used to detect the release of LMWF (Amin et al., 2021; Liu et al., 2022). First, 250 mL Milli-Q water was used to dissolve 4 mg DMMB dye. The obtained solution was mixed with glycine (0.75 g) and sodium chloride (400 mg), and the pH was adjusted to 3 by adding 1 N hydrochloric acid. Finally, the resultant solution was filtered through a sterile filter film (0.22 μm), removing contaminants. Utilizing a UV-visible spectrophotometer, the absorbance was measured at 525 nm, and the standard curve (0–200 μg/mL) was generated to estimate the LMWF concentration. The concentration of LMWF in the solution was calculated according to the standard curve (*r* ^2^ > 0.999) by taking 50 mg of the sample from each of the three groups of nanofiber membranes and measuring the absorbance at 525 nm after thorough stirring in 5 mL artificial saliva. The theoretical content of LMWF in the samples was calculated according to the ratio between the components.

$$\text{Drug}\;\text{loading}\;\text{efficiency}\left(‰\right)=\frac{W1}{W3}\times 1000$$ (2)

$$Encapsulation\;efficiency\left(\%\right)=\frac{W2-W1}{W2}\times 100$$ (3)

In the formula, W1 is the actual LMWF content; W2 is the theoretical LMWF content; W3 is the sample mass.

#### 2.13.8 *In vitro* drug release

Three sets of nanofiber membranes (10 mg) were placed in 5 mL of artificial saliva (pH = 6.8) and treated at a constant rate of 120 rpm at 37°C using a thermostatic shaker (HNY-200B, Honor, Tianjin, China). After removing 1 mL of solution at different time points, 1 mL of artificial saliva was added. The absorbance values (*n* = 3) were measured at 525 nm using a UV-Vis spectrophotometer, and the drug concentration and percentage cumulative drug release at each time point were calculated from the standard curve equation and analyzed using Origin 9.0 to plot the cumulative drug release curve.

#### 2.13.9 Degradation of nanofibrous membranes

To study the degradation of nanofiber membranes, nanofiber membranes were immersed in artificial saliva and treated at a constant rate of 120 rpm at 37°C using a thermostatic shaker for 3 days. Then, the surface morphology was observed by SEM after natural drying.

### 2.14 Statistical analysis

All data were expressed as mean ± standard deviation (x ± SD), and the experiments were statistically analyzed using the GraphPad Prism 9.0 software. Differences between two groups were compared using Student’s t-test, and differences between three or more data groups were analyzed using one-way ANOVA. Error bars indicate the SD of triplicate measurements for each group. Differences were considered statistically significant at *p* < 0.05.

## 3 Results and discussion

### 3.1 Impact of SR-A in the development of OLK occurrence

**FIGURE 1:**
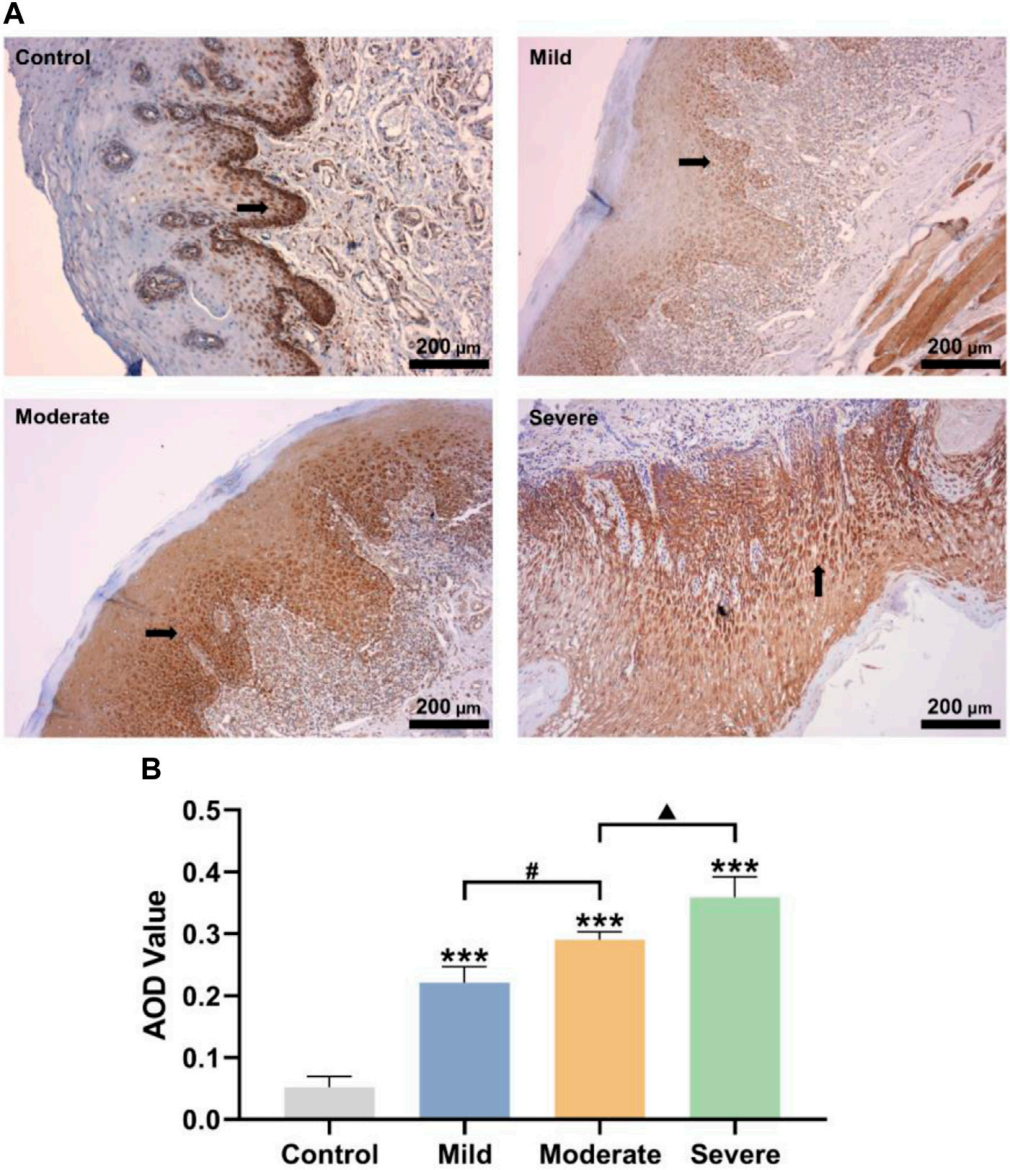
Impact of SR-A in the development of OLK occurrence. **(A)** Control: Representative pictures of oral epithelial immunohistochemistry in healthy people; mild, moderate, and severe: representative images of the oral epithelium of OLK patients with different degrees of abnormal hyperplasia. Brownish-yellow particles represented SR-A positive expressions. Scale bar = 200 μm. **(B)** AOD values of SR-A positive substances in control, mild, moderate, and severe groups. Data were expressed as the means ± SD. ***p < 0.001 vs. Control group; ^▲^ *p* < 0.05 vs. Moderate group; ^#^ *p* < 0.05 vs. Mild group.

OLK is the most prevalent oral potentially malignant disease (OPMD) with a global incidence of 4.1% (Aguirre-Urizar et al., 2021). Genes, biomarkers, and risk factors (such as smoking, drinking, and eating betel nuts) have been implicated in the development of OLK (Neville and Day, 2002; Farah, 2021). However, OLK has no valid prevention or treatment and is highly susceptible to recurrence or adverse reactions (Lodi et al., 2016). SR-A is expressed in various human cells and can activate the host’s innate immune response by recognizing the molecular patterns of different pathogens (PrabhuDas et al., 2017). To investigate the effect of SR-A on OLK, SR-A expression in normal mucosal and OLK tissues was examined using immunohistochemistry. The results showed that SR-A was expressed only in a small amount in the basal layer of the mucosal epithelium in the normal population, whereas it was expressed in most of the basal layer and a small portion of the granular layer of the mucosal epithelium of the mildly dysplastic mucosal epithelium; in the moderately dysplastic mucosal epithelium it was expressed in all of the basal layer and a portion of the granular layer; and it was expressed in large amounts throughout the entire epithelium of the severely dysplastic mucosal epithelium (Figure 1A). Figure 1B shows that the AOD values of SR-A-positive expression were significantly higher in the mild, moderate, and severe groups (0.221 ± 0.021, 0.29 ± 0.011, and 0.358 ± 0.027, respectively) when compared to the control group (0.052 ± 0.014). The immunohistochemical results illustrated that SR-A in the oral mucosal epithelium was abnormally highly expressed in patients with OLK compared to the normal population and increased with the degree of abnormal oral epithelial hyperplasia. SR-A may be involved in the malignant transformation of the oral epithelium, influencing and promoting the development of OLK.

### 3.2 Establishment of the SR-A RNAi model and the effect of SR-A on DOK cells

**FIGURE 2:**
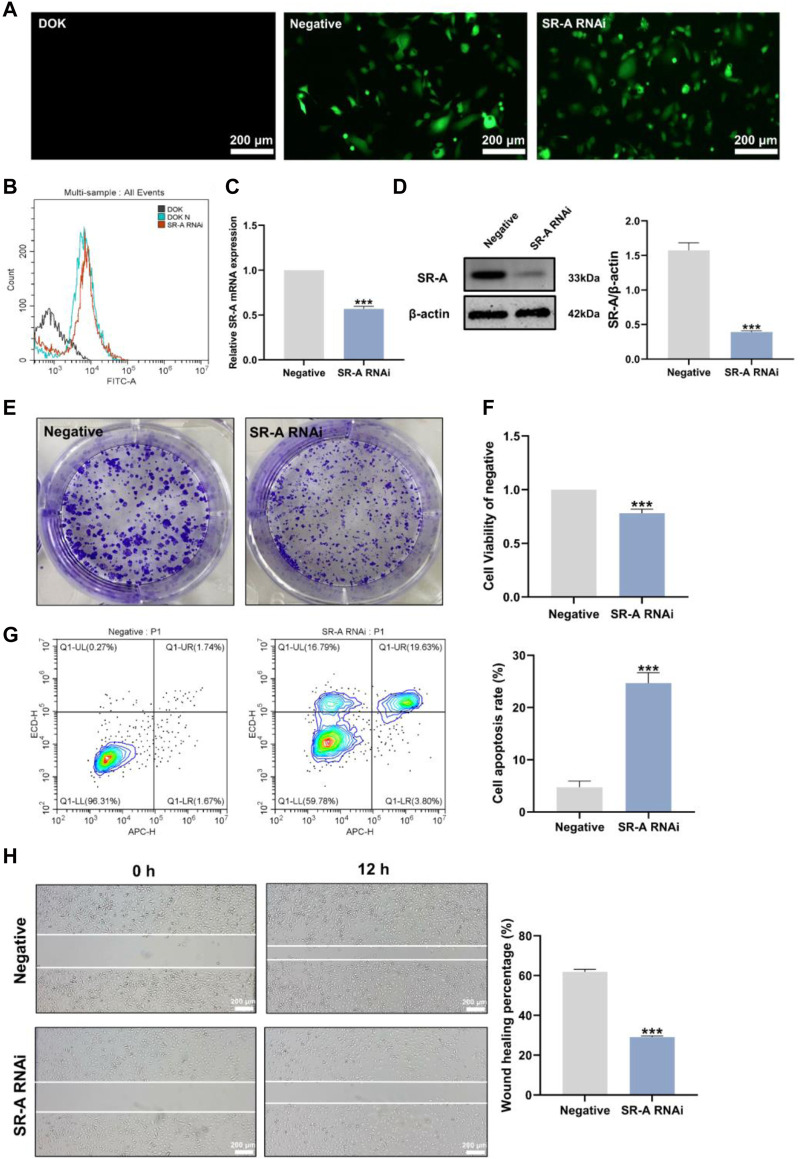
Establishment of the SR-A RNAi model and the effect of SR-A on DOK cells. **(A)** Representative images of lentiviral transfection efficiency. Green fluorescence indicates lentiviral vector particles. Scale bar = 200 μm. **(B)** Lentiviral transfection efficiency by flow cytometry assay. **(C)** and **(D)** The mRNA and protein expression levels of SR-A. **(E)** Colony-forming ability of DOK negative cells and SR-A RNAi groups **(F)** Cell viability in DOK negative cells and SR-A RNAi groups **(G)** Apoptosis rates in DOK negative cells and SR-A RNAi groups **(H)** Migration ability in DOK negative cells and SR-A RNAi groups. Scale bar = 200 μm. Data were expressed as the means ± SD. ***p < 0.001 vs. DOK negative group.

RNA interference-knockdown of target genes (RNAi) has been used to develop new drugs and treat illnesses (Brioschi and Banfi, 2018). To explore whether SR-A is a target site for the regulation of OLK development, we established an SR-A RNAi model by transfecting lentiviral vectors. Fluorescence microscopy revealed that the SR-A RNAi and negative groups had a significant quantity of green fluorescence present in nearly every cell compared to the untransfected DOK cells, which exhibited no green fluorescence (Figure 2A). Flow cytometry assays showed enhanced FITC signaling in the SR-A RNAi- and SR-A-negative groups compared to that in untransfected DOK cells (Figure 2B). The results indicated that the SR-A RNAi lentiviral particles were successfully transfected with high transfection efficiency. The SR-A mRNA and protein expression levels were considerably decreased in cells after knockdown (Figures 2C, D). All the above findings showed that the SR-A RNAi model was successfully established and might be utilized in further research to confirm the impact of SR-A on DOK cells.

SR-A, also known as macrophage SR 1 (MSR1), is primarily found on the surface of different types of macrophages and is critical for macrophage M2 polarization (Labonte et al., 2017). It is involved in several diseases and processes, including cancer (Gudgeon et al., 2022). According to previous studies, the degree of macrophage M2 polarization is linked to the development of OLK (Weber et al., 2020; Zhu Y. et al., 2021). However, the precise effects of SR-A on OLK remain unknown. Therefore, we employed SR-A RNAi as the experimental group and DOK-negative cells as the control group and conducted correlation experiments on both cell types to clarify the effect of SR-A on OLK. Compared to the control group, DOK cells with SR-A knockdown showed considerably reduced vitality and capacity for cell colony formation (Figures 2E, F). Additionally, the cell scratch experiment demonstrated that after knocking down the SR-A gene, the migration capacity of DOK cells was diminished compared to that of the control group (Figure 2H). The flow assay was used to measure the apoptosis level in both groups, and compared to the control group, the apoptosis rate of DOK cells was considerably higher after SR-A knockdown (Figure 2G). These results suggest that SR-A knockdown may inhibit DOK growth and promote DOK apoptosis, which then slows down the development of OLK, demonstrating that SR-A could act as a critical factor in regulating OLK.

### 3.3 General property analysis of LMWF

**FIGURE 3:**
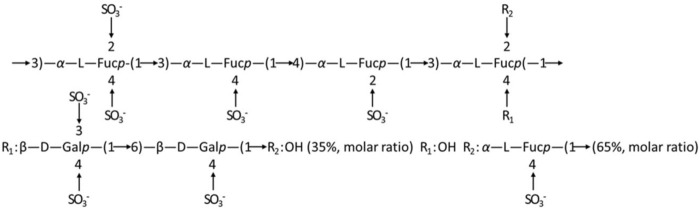
Speculation on the structure of fucoidan sulfate in *Laminaria japonica* (Wang et al., 2010).

LMWF is a complex water-soluble sulfated polysaccharide with a large molecular weight that is extracted from *Laminaria japonica* (Wang et al., 2019). It has strong biological activities such as anti-inflammatory and anti-cancer properties (Sanjeewa et al., 2017; Cui et al., 2022). The relevant chemical properties and structure of LMWF were first analyzed. With a molecular weight of 8,177 Da, LMWF had a high content of fucose (35.07%) and sulfate (36.85%) and a low content of glyoxylate (0.039%). Fucose (Fuc) and galactose (Gal) comprised most of the monosaccharides (Table 1). Second, the structure of LMWF was characterized in a previous study (Wang et al., 2010). As shown in Figure 3, it was different from known fucoidans. LMWF, which infers to LF2 in previous studies, contained more (1 → 3) linkages than (1 → 4) linkages. The sulfate groups were distributed non-uniformly and attached to C-3 and C-4 of galactose rather than to C-2 and C-4 of fucose (Wu et al., 2021).

**TABLE 1:** Chemical composition analysis (%, dry weight) of LMWF.

Sample	Sulfate (%)	Fucose (%)	Uronic acid (%)	Mw (Da)	Neutral monosaccharide composition (molar ratio)			
					Fuc	Gal	Xyl	Glc
LMWF	36.85	35.07	0.039	8,177	1.000	0.094	0.026	0.015

### 3.4 Effect of LMWF on DOK cells

**FIGURE 4:**
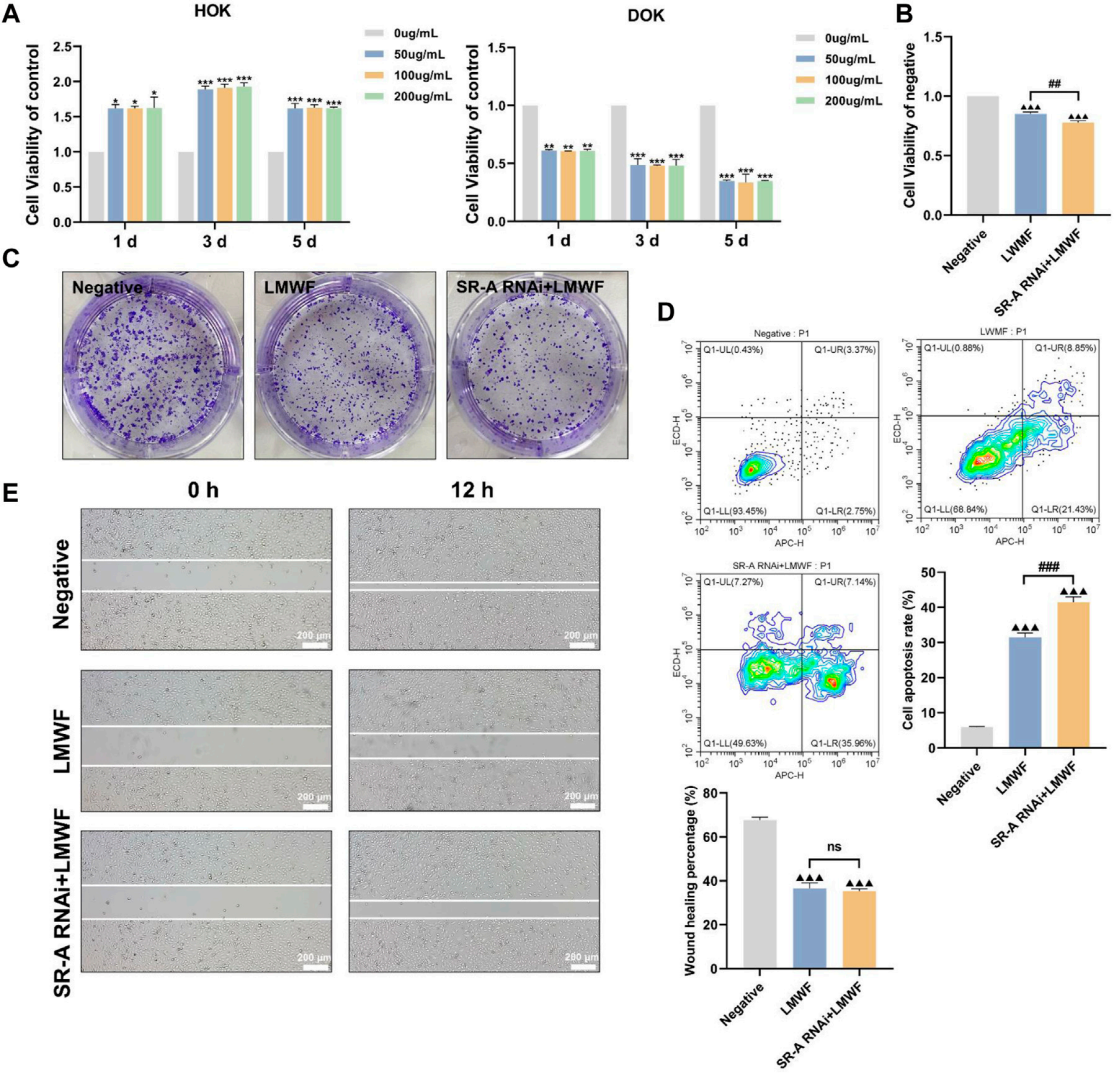
Effect of LMWF on DOK cells. **(A)** Effect of different concentrations of LMWF on the viability of DOK and HOK cells **(B)** Proliferative capacity of cells **(C)** Colony-forming ability of different groups **(D)** Apoptosis rates of cells **(E)** Migration ability of cells. Scale bar = 200 μm. Data were expressed as the means ± SD. ***p < 0.001, **p < 0.01 and *p < 0.05 vs. 0 μg/mL group; ^▲▲▲^ *p* < 0.001 vs. DOK negative group; ^###^ *p* < 0.001 and ^##^ *p* < 0.01 vs. LMWF group.

Owing to its unique structure, LMWF is a natural ligand for various proteins such as SR-A and selectin (Zhu X. et al., 2021). We have previously found that LMWF inhibits SR-A-mediated lipid uptake and activates multiple signaling pathways to facilitate the development of atherosclerosis (Xu et al., 2019; Sun et al., 2022). These results confirm that SR-A influences and promotes the development of OLK. Therefore, it is speculated that the targeted binding of LMWF to SR-A may block the action of OLK. CCK8 trials revealed the effects of LMWF at different concentrations (0 μg/mL, 50 μg/mL, 100 μg/mL, and 200 μg/mL) on DOK and HOK cells (Bi et al., 2018). Figure 4A shows that LMWF had a significantly inhibitory effect on DOK cells compared with the control group at 50–200 μg/mL, whereas there was no toxic effect on HOK cells. According to the principle of the drug dose–effect relationship, LMWF at a concentration of 50 μg/mL was selected as the concentration for subsequent experiments.

To further investigate whether the inhibitory effect of LMWF on DOK cells could be achieved by regulating the SR-A gene, DOK-negative and SR-A RNAi cells were treated with LMWF separately. DOK-negative cells without LMWF served as the control group. Compared to the control group, DOK-negative and SR-A RNAi cells treated with LMWF were considerably less viable and had an insufficient ability to form colonies (Figures 4B, C). Additionally, compared with the control group, the cell scratch assay revealed that DOK-negative and SR-A RNAi cells were less able to migrate after LMWF treatment (Figure 4E). Flow cytometry results revealed a statistically significant increase in apoptosis rates in both groups when LMWF was added, compared to the control group (Figure 4D). These results suggest that LMWF promotes the apoptosis of DOK cells and inhibits their proliferation and migration. This indicates that LMWF has a potential therapeutic effect in inhibiting the development of OLK, and that the inhibitory effect of LMWF might be achieved through the regulation of SR-A.

### 3.5 Effect of LMWF on related signaling pathways and genes

**FIGURE 5:**
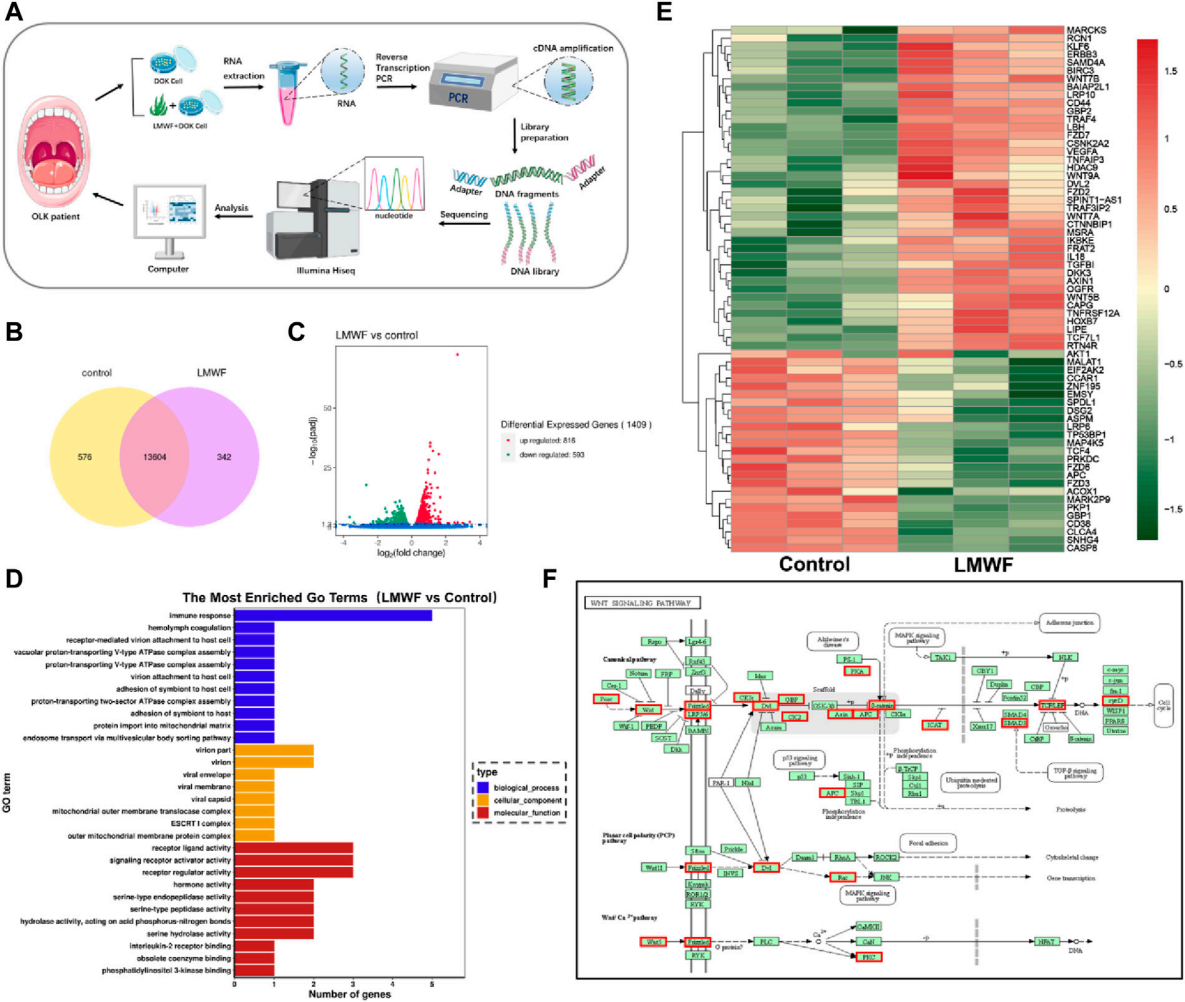
Effect of LMWF on related signaling pathways and genes. **(A)** Schematic diagram of the high-throughput sequencing process. **(B)** Venn diagram of differentially expressed genes (FPKM > 1). **(C)** Gene differential volcano plot. Red dots (upregulated) and green dots (downregulated) indicated genes with significant differential expression. The horizontal coordinates represented gene expression fold changes in different samples, and the vertical coordinates represented the statistical significance of the differences in gene expression changes. Blue dots denoted genes with negligible differential expression. **(D)** The enriched GO term was the vertical coordinate, and the number of differentially expressed genes in that word was the horizontal coordinate. Different colors denote various cellular components, molecular activities, and biological processes. **(E)** Map of the differential gene clustering. Each row represented one gene, and each column represented one sample. The hue changes from red to green, indicating that lg (FPKM+1) went from large to tiny. **(F)** Diagram of the metabolic pathway considerably enriched in the KEGG database; red boxes indicated differential genes.

LMWF regulates multiple signaling pathways (Xu et al., 2019). To further elucidate the regulation mechanism of the LMWF target SR-A gene to block the development of OLK, DOK cells were cultured using a culture medium with LMWF (50 μg/mL) and an equal volume of culture medium for 24 h. Transcriptomes were then subjected to high-throughput sequencing (Figure 5A). Venn diagrams and volcano mapping revealed many differentially expressed genes between the LMWF and control groups (Figures 5B, C). Gene ontology (GO) functional enrichment analysis showed that various genes were involved in immune response, receptor-ligand activity, and receptor regulator activity (Figure 5D). However, the heat map and enriched KEGG pathway map revealed that LMWF impacted some Wnt signaling pathway-related factors, including Frizzled, APC, and GBP, as well as certain inflammatory factors such as TNF-α and TGF-β (Figures 5E, F). During evolution, the Wnt signaling pathway is a highly conserved signaling pathway that regulates cell proliferation, apoptosis, differentiation, migration, and genetic stability. This has potential implications for the regulation of immune responses in the tumor microenvironment (Pai et al., 2017). This pathway also involves different degrees of abnormal hyperplasia and malignant transformation in oral dysplasia (Reyes et al., 2020; Xie et al., 2020). It was shown that the Wnt pathway has an essential role in the malignant process of oral diseases such as OLK, oral mucosal fibrosis, oral lichen planus, and oral erythema (Ejaz and Ghafoor, 2019), and its classical pathway-related components WNT3 and β-catenin are overexpressed in OLK epithelial cells (Ishida et al., 2007), suggesting that this pathway may be involved in the abnormal hyperplasia of OLK oral epithelium (Zhang et al., 2017; Bhattacharyya et al., 2021).

### 3.6 Effect of LMWF on SR-A/Wnt signaling axis and related gene expression

**FIGURE 6:**
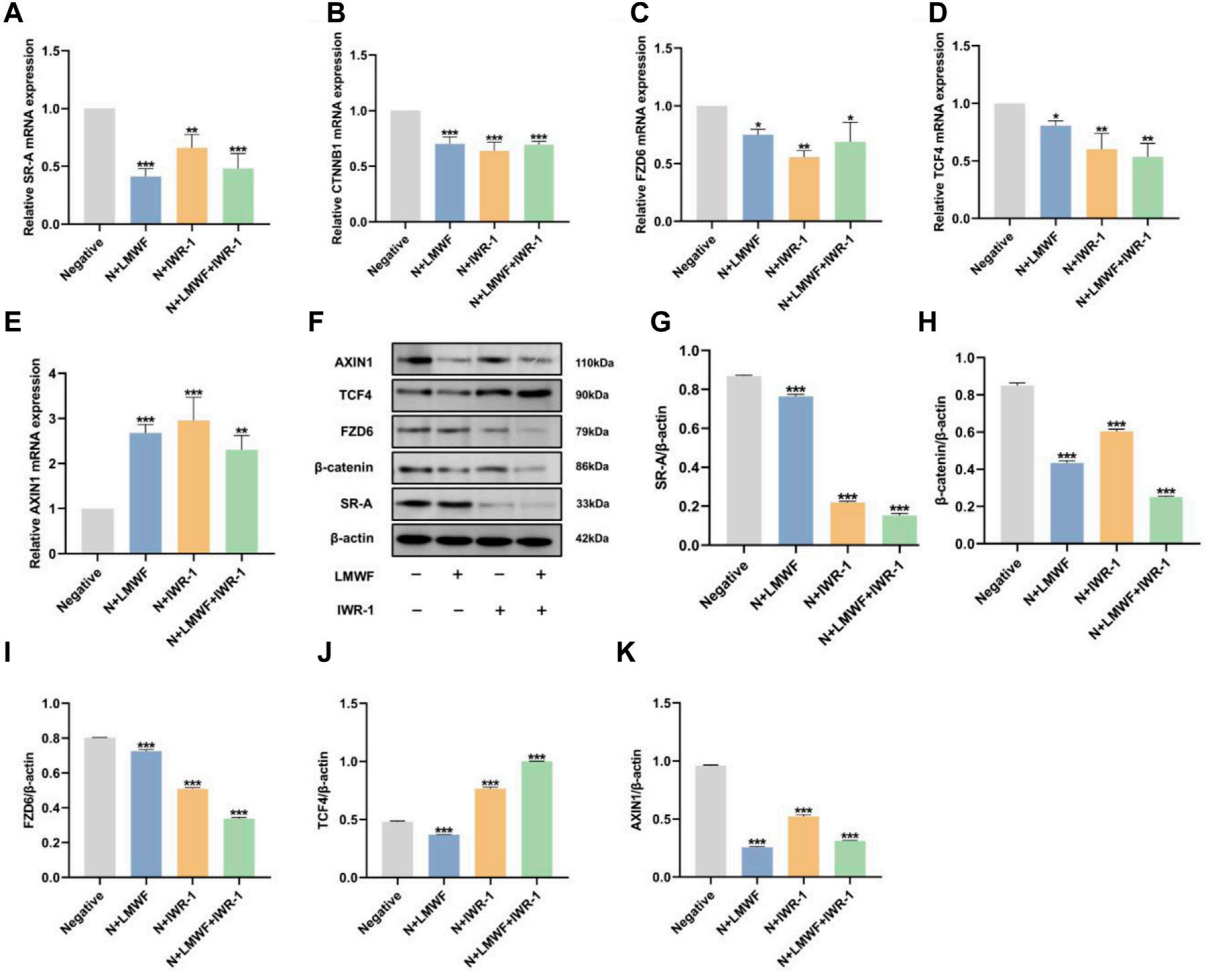
Effect of LMWF on the SR-A/Wnt signaling axis and related gene expression. **(A–E)** After different treatments, the expression of mRNA for SR-A, CTNNB1, FZD6, TCF4, and AXIN1. **(F)** Representative protein expression bands for SR-A, CTNNB1, FZD6, TCF4, AXIN1, and β-actin. **(G–K)** Relative expression levels of SR-A, CTNNB1, FZD6, TCF4, and AXIN1 proteins compared to β-actin. Data were expressed as the means ± SD. ***p < 0.001, **p < 0.01 and *p < 0.05 vs. DOK negative group.

Based on the previous results and the malignant transformation role of Wnt in OLK, to explore the effects and mechanism of LMWF on the SR-A/Wnt signaling axis, IWR-1 was selected as an inhibitor of the Wnt signaling pathway. It could abort the turnover of Axin to form the β-catenin disruption complex and then inhibit the activity of the Wnt classical pathway (Chen et al., 2009). The expression levels of SR-A-related proteins and the Wnt signaling pathway were detected using RT-qPCR and WB. The results showed that the mRNA levels of SR-A, CTNNB1, TCF4, and FZD6 decreased and that of AXIN1 increased after LMWF treatment (Figures 6A–E). These results were consistent with those of high-throughput sequencing. This indicated that LMWF affected the expression of SR-A and Wnt pathway-related genes to different degrees. After treatment, the mRNA levels of both the SR-A and Wnt signaling pathway-related genes were significantly altered. The variation in mRNA levels in the LMWF + IWR-1 group was similar to that in the LMWF and IWR-1 groups. Therefore, the above results indicate that LMWF can reduce the expression of SR-A and has different influences on the upstream and downstream genes of the Wnt pathway, suggesting that the Wnt-related gene pathway might be involved in the regulation of the SR-A gene by LMWF.

The WB results showed that the protein expression levels of SR-A, CTNNB1, TCF4, and FZD6 were downregulated in the LMWF group compared to those in the control group, which is consistent with the RT-qPCR results. The protein expression levels of SR-A, CTNNB1, and FZD6 were downregulated in the IWR-1 and LMWF + IWR-1 groups, similar to those in the LMWF group (Figures 6G–I). However, the level of TCF4 protein expression in the IWR-1 and IWR-1+LMWF groups was opposite to that of gene transcription (Figures 6F, J). This may be related to the function of TCF4. TCF4 is the most critical transcription factor in the TCF/LEF family that binds to β-catenin to regulate transcription of Wnt target genes (Wang Y. et al., 2022). However, several other members of the TCF family (TCF1, LEF, and TCF3) can activate the Wnt signaling pathway via TCF4 (van Es et al., 2012; Wallmen et al., 2012). Therefore, the addition of IWR-1 may affect the different “wiring” of the intracellular signaling mechanism, resulting in other TCF/LEF families undertaking the role of TCF4. This resulted in increased TCF4 protein levels in the IWR-1 and IWR-1+LMWF groups (Figures 6F, J). Surprisingly, the overall protein expression level of AXIN1 was significantly downregulated compared to that in the control group, in complete contrast to the RT-qPCR results (Figures 6F, K). This may be due to the role of AXIN1 in the formation of degradative complex bodies induced by tankyrase-associated inhibitors, which remains unclear. Relevant studies have shown that AXIN1 is not required for the formation of degradative complex bodies, and it may predominate in mediating the transition from Wnt-off to Wnt-on a state by being recruited to signalosomes (Leung et al., 2002; Thorvaldsen et al., 2017); thus, its role may not be prominent at the protein level. In conclusion, these results suggest that LMWF regulates the SR-A/Wnt signaling axis and related gene expression to achieve a therapeutic effect against OLK.

### 3.7 Construction and surface morphology of LMWF/PLCL nanofiber membranes

**FIGURE 7:**
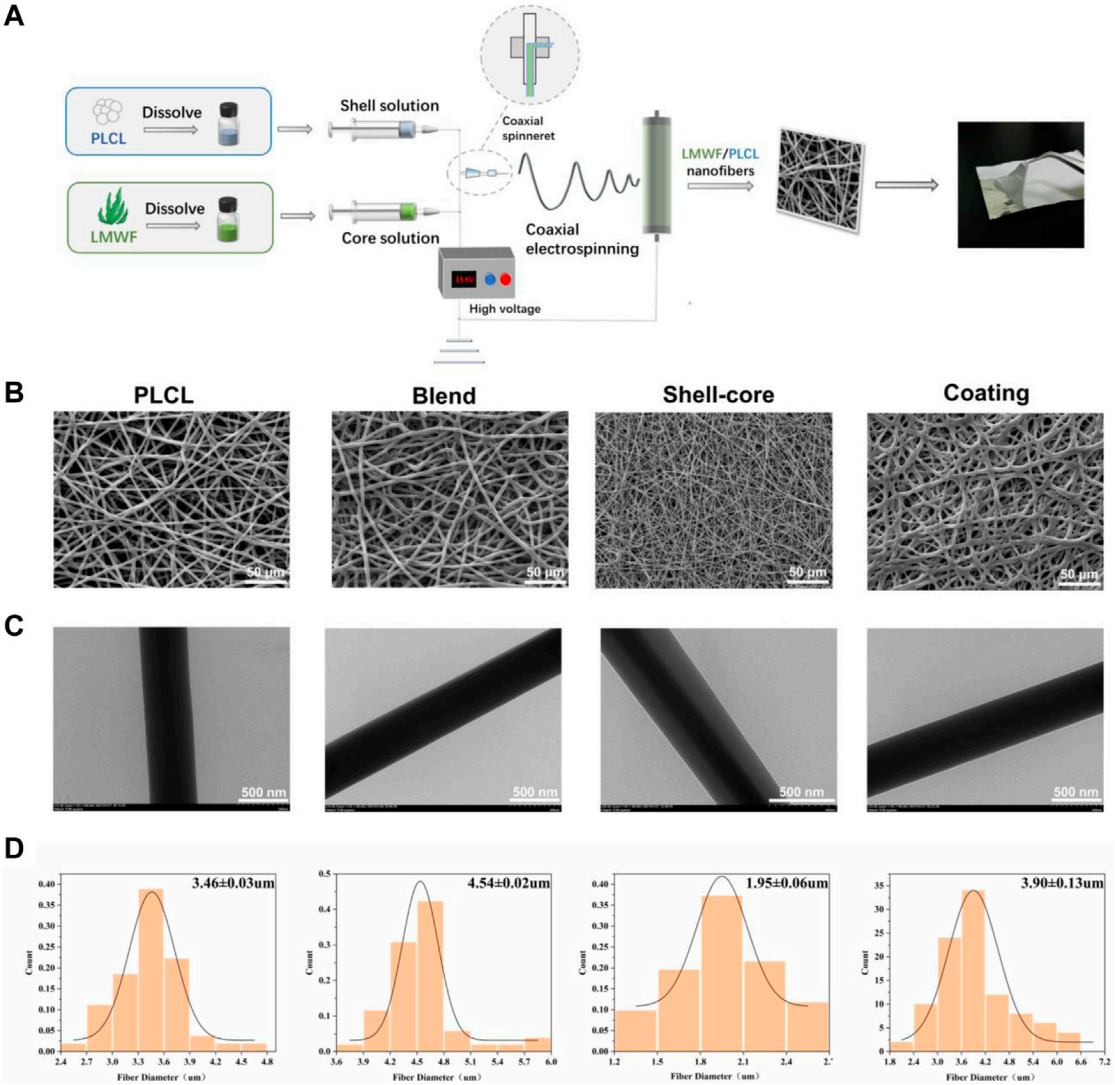
Construction and surface morphology of LMWF/PLCL nanofiber membranes. **(A)** Schematic diagram of nanofiber membrane spinning process. **(B)** SEM images of different nanofiber membranes. Scale bar = 50 μm. **(C)** TEM images of different nanofiber membranes. Scale bar = 500 nm. **(D)** Diameter distribution of different nanofibers.

Owing to the special characteristics of the oral environment, saliva and diet can affect the effectiveness of drug treatments for OLK lesions (Wang X. et al., 2022). As LMWF is a water-soluble heteropolysaccharide that is rapidly diluted in the oral cavity (Tyeb et al., 2023), resulting in drug loss, traditional therapeutic approaches, such as topical and oral administration, cannot ensure effective drug concentration at the lesion site. Electrospinning, an efficient drug delivery technique, has become increasingly popular in recent years for the topical treatment of oral problems (Edmans et al., 2020; Sadeghi et al., 2020). To achieve optimal drug delivery, we developed novel LMWF/PLCL nanofiber membranes using different electrospinning techniques (Figure 7A). According to the SEM images, the nanofibers exhibited an interwoven mesh structure without a bead-like structure, with smooth surfaces, uniform thickness, and random distribution (Figure 7B). The TEM images showed that the shell-core nanofiber membrane had an obvious shell-core structure. The drug was encapsulated in the shell-layer material with an excellent drug-carrying effect (Figure 7C). These results indicate that the spinning solution with added LMWF can successfully spin nanofiber membranes with no significant impact on the morphology of the nanofiber surface. After evaluating the nanofiber diameter size (n ≥ 50) with ImageJ, the addition of LMWF was found to have a specific effect on the diameter of the nanofibers. The fiber diameters of the blended and coated nanofiber membranes became coarser, whereas the diameter of the shell-core nanofiber membranes decreased (Figure 7D).

### 3.8 Physicochemical characterization of LMWF/PLCL nanofiber membranes

**FIGURE 8:**
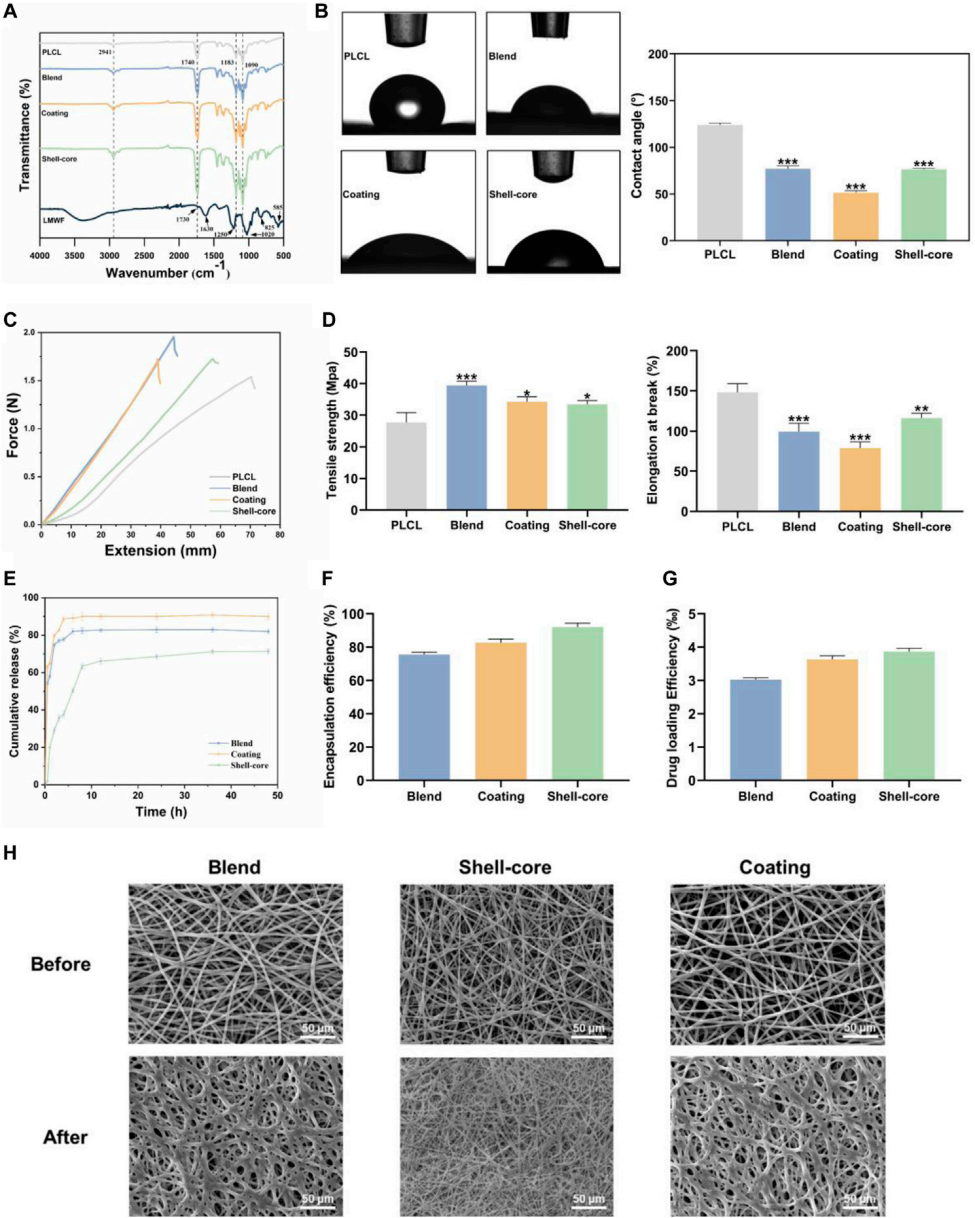
Physicochemical characterization of LMWF/PLCL nanofiber membranes. **(A)** FTIR spectra of LMWF and nanofiber membranes. **(B)** WCA images and analytical plots of different nanofiber membranes at 10 s. **(C)** Schematic diagram of the mechanical properties of nanofiber membranes. **(D)** Tensile strength and elongation at break of nanofiber membranes. **(E)** Cumulative drug release curves of nanofiber membranes. **(F)** Encapsulation rate of nanofiber membranes. **(G)** The drug loading rate of nanofiber membranes. **(H)** The change of fiber morphology after LMWF/PLCL nanofiber membranes were soaked in artificial saliva. Scale bar = 50 μm ^***^ *p* < 0.001, **p < 0.01 and *p < 0.05 vs. PLCL group.

To further verify whether the nanofibrous membranes can be adapted to a particular drug delivery environment in the oral cavity, we conducted physicochemical characterization to comprehensively screen out the best nanofibrous membranes suitable for the oral mucosa. The functional groups and intermolecular interactions in the LMWF and nanofiber membranes were determined by analyzing their FTIR spectra. Figure 8A shows that LMWF exhibited characteristic peaks at 1730 cm^−1^ (CO stretching band of acetyl group), 1,630 cm^−1^ (C-O-O antisymmetric stretching band of glucuronide carboxylate group), 1,250 cm^−1^ (S=O stretching band of sulfate group), 1,020 cm^−1^ (C-O-C symmetric stretching band of glycoside group), 825 cm^−1^ (C-O-S stretching band of sulfate group), and 585 cm^−1^ (S=O bonded stretching band of sulfate group) (Saravana et al., 2018; Fernando et al., 2020). For PLCL, the representative absorption peaks at 1740 cm^−1^, 1,183 cm^−1^, and 1,090 cm^−1^ correspond to -COOR and C-O stretching vibrations, and the other unique peak at 2,941 cm^−1^ represents the stretching vibrations of -CH2 and -CH3 (Khan et al., 2019; Wang S. et al., 2022). The characteristic peaks associated with LMWF in the LMWF/PLCL nanofiber membranes may be due to the low concentration of LMWF in the nanofibers, or the characteristic peaks of LMWF may be obscured by the characteristic peaks of PLCL. Compared to the control group, the absorption peaks of the nanofiber membrane at 1,090 cm^−1^, 1,183 cm^−1^, 1740 cm^−1^, and 2,941 cm^−1^ gradually increased with the addition of the drug, and the different degrees of increase may be related to the difference in actual drug loading. As no new peaks or significant shifts appeared, there was no chemical interaction between LMWF and PLCL, and the nature of the interaction between the two substances was physical. This proves that LMWF was successfully loaded onto the nanofiber membrane.

Biocompatibility is closely related to the hydrophobicity of the nanofiber membranes. The hydrophilicity of the nanofiber membranes creates conditions for early cell attachment, making it easier for the drug to interact with the cells (Li et al., 2012). WCA data showed that the contact angle of the PLCL nanofiber membrane was 124.03 ± 1.47°. This is because of the high proportion of hydrophobic groups in polymers. The absence of hydrophilic groups makes the surface of the substance extremely hydrophobic, adversely affecting its affinity for cells. However, the hydrophilicity of the nanofiber membrane increased with the addition of LMWF (Figure 8B). The use of composite systems with synthetic and natural polymers provides superior hydrophilicity compared to synthetic polymers alone because of the role of functional groups, such as hydroxyl, carboxyl, amine, and sulfate groups, which are frequent in natural polymers (Chandrasekaran et al., 2011). The hydrophilicity of the nanofiber membrane was significantly improved by the presence of sulfate and hydroxyl groups in LMWF (Lee et al., 2012; Cunha and Grenha, 2016). The plasma modification of the surface, which increased the hydrophilicity of the nanofiber membrane surface to increase the physical adsorption effectiveness (Laurano et al., 2019; Al-Dhahebi et al., 2020), resulted in the coated nanofiber membrane’s best hydrophilicity of 51.34 ± 1.81°. In summary, the three groups of nanofiber membranes were more hydrophilic, which was conducive for adhesion to the oral mucosa.

The integrity and tensile strength of oral patches are critical when they come in contact with the mucosal tissues surrounding the oral cavity (Goyal et al., 2018). Based on tensile testing, the tensile strength of PLCL nanofiber film was 27.7 ± 2.55 MPa, and the elongation at break was 148.1% ± 8.82%. With the addition of LMWF, the tensile strength of the LMWF/PLCL nanofiber membrane increased significantly and the elongation at break decreased (Lee et al., 2012). The tensile strengths of blended, coated, and shell-core nanofiber membranes were 39.42 ± 1.1 MPa, 34.28 ± 1.27 MPa, and 33.45 ± 0.99 MPa, respectively, and the elongations at break were 99.63% ± 8.82%, 78.83% ± 8.09%, and 116.03% ± 6.36%, respectively (Figures 8C, D). The addition of natural polymers, such as collagen and chitosan, to synthetic polymers, such as PLCL and PCL, increased their tensile strength and decreased their elongation at break (Lee et al., 2008; Li et al., 2012). In conclusion, the mechanical properties of nanofibrous membranes were improved by adding LMWF, and the shell-core nanofibrous membranes maintained a high tensile strength and elongation at break, with adequate strength and flexibility to adapt to the unique oral environment.

The therapeutic action of pharmaceuticals at the lesion site depends on the effectiveness of drug encapsulation and the drug-loading capacity of the nanofibrous membranes. It can be observed from Figures 8F,G that different electrospinning preparation methods significantly affected the drug-loading and encapsulation rates, and the trends of the drug-loading and encapsulation rates of the three groups of nanofiber membranes were consistent. The drug-loading rate of the blended nanofiber membrane was 3.02‰ ± 0.05‰, and the encapsulation rate was 75.56% ± 1.13%, the lowest among the three groups. The drug-loading rate of the shell-core nanofiber membrane was 3.87‰ ± 0.08‰, and the encapsulation rate was 92.04% ± 1.93%, the highest among the three groups. Shell-core nanofiber membranes have a unique shell-core structure, and the drug can be well wrapped in the core layer, resulting in maximum drug-loading and encapsulation rates and a corresponding increase in drug utilization (Li J. et al., 2022). This suggests that LMWF can be successfully encapsulated in nanofibers using the electrospinning technique, and both the drug-loading rate and encapsulation efficiency can be improved.

The ability of nanofiber membranes to slow drug release is critical for maintaining effective drug concentrations at the lesion site. As shown in the drug release profile (Figure 8E), the drug release from the nanofiber membranes varied widely. Within 2 h, the degree of sudden release of the blended and coated nanofiber membrane drug was drastic, with cumulative release reaching 74.89% ± 1.01% and 79.74% ± 1.15%, respectively, followed by a slow-release phase. For the shell-core nanofiber membrane, because the core drug was wrapped by the shell layer, the degree of sudden drug release was more moderate, and the cumulative slow-release rate of the drug in the sudden-release stage was 50.41% ± 1.02%, which then entered into the slow-release phase. The cumulative sustained release rate at 48 h was 71.33% ± 1.15%, which was the best-sustained release because there was still ample space for drug release.

In addition, the degradation of LMWF/PLCL nanofiber membranes were tested and analyzed. After soaking in artificial saliva for 3 days, the surface morphology of the nanofiber membranes was observed by scanning electron microscope (Figure 8H). As expected, the nanofibers are partially dissolved because LMWF is dissolved in the nanofiber membranes. The undissolved part is PLCL, which acts as a mechanical support. The degradation quality did not change obviously. Nanofibers provide great flexibility in the selection of materials for drug delivery applications. The polymer composition of nanofiber membrane is the key to determine the kinetics of drug release (Jiffrin et al., 2022). Both biodegradable and non-degradable materials can control the release rate of drugs through diffusion and degradation.

Based on the evaluation of the physicochemical properties of the three groups of nanofibrous membranes mentioned above, the nanofibrous membranes with a shell-core structure could satisfy the unique drug delivery environment of the oral cavity and ensure the effective drug concentration at the OLK lesion site. Therefore, they were selected for the subsequent experiments.

### 3.9 Evaluation of biocompatibility and treatment efficacy of LMWF/PLCL nanofiber membranes

**FIGURE 9:**
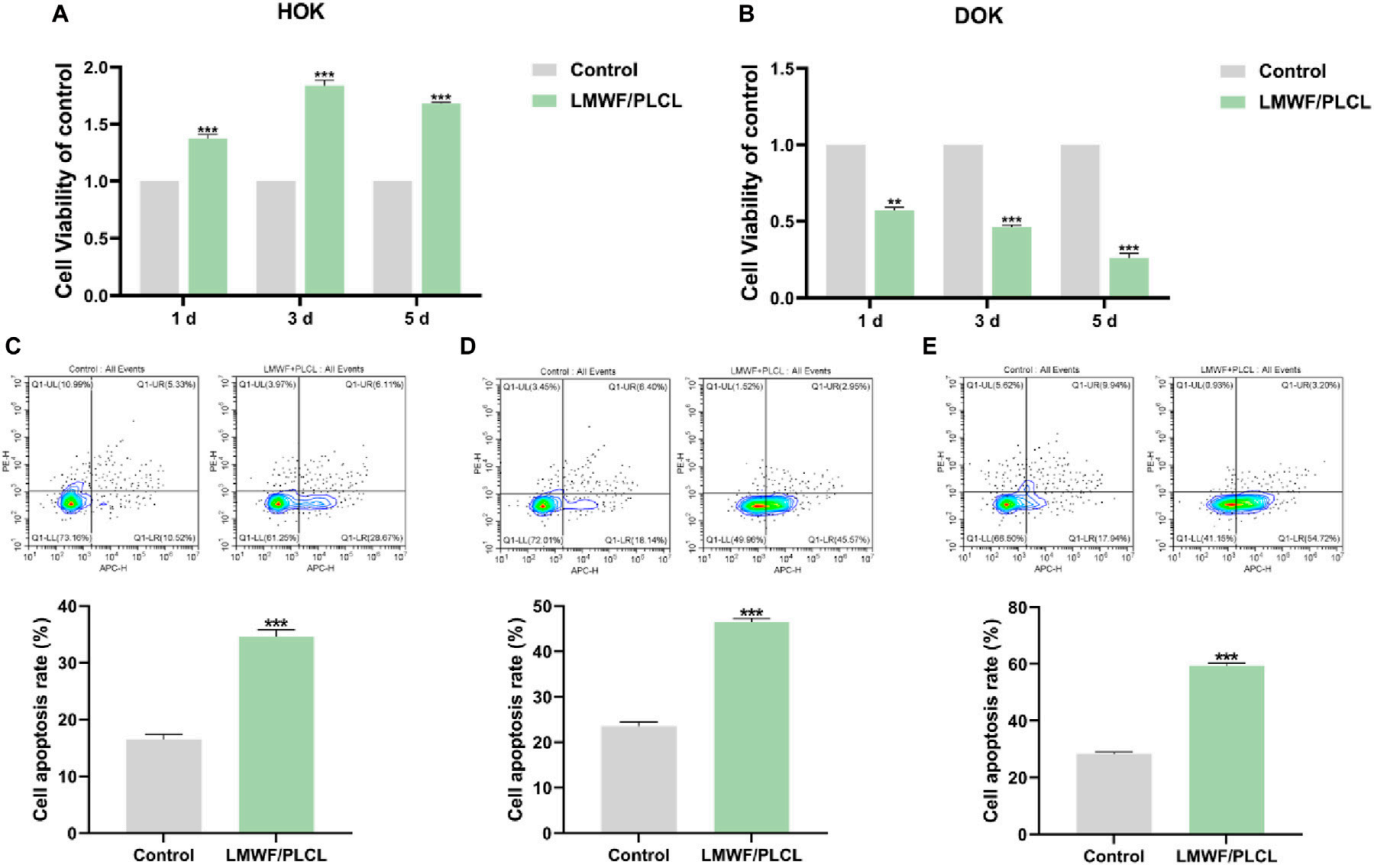
Evaluation of biocompatibility and treatment efficacy of LMWF/PLCL nanofiber membranes. **(A)** Effect of nanofiber membranes on HOK cell viability. **(B)** Effect of nanofiber membranes on DOK cell viability. **(C–E)** The apoptosis rate of DOK cells co-cultured with nanofibrous membrane for 1, 3, and 5 days. ***p < 0.001, **p < 0.01 vs. Control group.

Biocompatibility is one of the most important considerations in the evaluation of biomedical materials (Liu et al., 2023). Therefore, we co-cultured HOK cells with nanofiber membranes for 1, 3, and 5 d to evaluate their biocompatibility via the CCK8 assay. As shown in Figure 9A, the LMWF/PLCL group exhibited no significant toxicity to HOK cells compared to the control group, indicating that the nanofiber membrane had good cytocompatibility. Similarly, the results of the CCK8 assay showed that the nanofiber membrane significantly inhibited DOK cell growth (Figure 9B). Flow cytometry results showed that the apoptosis rate of the LMWF/PLCL group increased with time, reaching 18.93%, 23.98%, and 30.04% at 1, 3, and 5 d, respectively (Figures 9C–E). Based on the above findings, it can be concluded that the LMWF/PLCL nanofiber membrane has good biocompatibility, and that the LMWF released from this drug delivery system has the same therapeutic effect as LMWF alone, but with a longer-lasting effect. This suggests that the nanofiber membrane can slowly release LMWF and maintain the effective concentration of the drug in the oral lesion for an extended period without being destroyed by saliva or food and without the need for repeated administration, thus enabling long-term delivery of the drug in the oral environment.

## 4 Conclusion

In summary, this study demonstrated that SR-A has a significant promotional role in the development of OLK, and that LMWF can inhibit DOK cells by regulating the SR-A/Wnt signaling axis. Thus, LMWF has the potential to be developed as a drug to prevent and treat OLK. In addition, the LMWF/PLCL nanofiber membrane of the novel shell-core structure was successfully developed for the special oral environment using electrospinning technology, which had better hydrophilicity, mechanical strength, drug-loading and encapsulation rates, drug release rate, and good biocompatibility and bioactivity. It maintained a high concentration of the effective drug at the lesion site in the special oral environment and promoted the apoptosis of DOK cells. Therefore, the novel shell-core-structured LMWF/PLCL nanofiber membrane may be used as a therapeutic oral patch for OLK.

## Data Availability

The original contributions presented in the study are included in the article/Supplementary material, further inquiries can be directed to the corresponding authors.
